# Satisfactory long-term clinical outcomes after bone marrow stimulation of osteochondral lesions of the talus

**DOI:** 10.1007/s00167-021-06630-8

**Published:** 2021-06-29

**Authors:** Quinten G. H. Rikken, Jari Dahmen, Sjoerd A. S. Stufkens, Gino M. M. J. Kerkhoffs

**Affiliations:** 1 Department of Orthopaedic Surgery, Amsterdam Movement Sciences, Amsterdam UMC, Location AMC, University of Amsterdam, Meibergdreef 9, 1105 AZ Amsterdam, The Netherlands; 2 Academic Center for Evidence Based Sports Medicine (ACES), Amsterdam UMC, Meibergdreef 9, 1105 AZ Amsterdam, The Netherlands; 3 Amsterdam Collaboration for Health and Safety in Sports (ACHSS), International Olympic Committee (IOC) Research Center, Amsterdam UMC, Meibergdreef 9, 1105 AZ Amsterdam, The Netherlands

**Keywords:** OLT, Osteochondral, Bone marrow stimulation, Talus, Long-term

## Abstract

**Purpose:**

The purpose of the present study was to evaluate the clinical and radiological outcomes of arthroscopic bone marrow stimulation (BMS) for the treatment of osteochondral lesions of the talus (OLTs) at long-term follow-up.

**Methods:**

A literature search was conducted from the earliest record until March 2021 to identify studies published using the PubMed, EMBASE (Ovid), and Cochrane Library databases. Clinical studies reporting on arthroscopic BMS for OLTs at a minimum of 8-year follow-up were included. The review was performed according to the PRISMA guidelines. Two authors independently conducted the article selection and conducted the quality assessment using the Methodological index for Non-randomized Studies (MINORS). The primary outcome was defined as clinical outcomes consisting of pain scores and patient-reported outcome measures. Secondary outcomes concerned the return to sport rate, reoperation rate, complication rate, and the rate of progression of degenerative changes within the tibiotalar joint as a measure of ankle osteoarthritis. Associated 95% confidence intervals (95% CI) were calculated based on the primary and secondary outcome measures.

**Results:**

Six studies with a total of 323 ankles (310 patients) were included at a mean pooled follow-up of 13.0 (9.5–13.9) years. The mean MINORS score of the included studies was 7.7 out of 16 points (range 6–9), indicating a low to moderate quality. The mean postoperative pooled American Orthopaedic Foot and Ankle Society (AOFAS) score was 83.8 (95% CI 83.6–84.1). 78% (95% CI 69.5–86.8) participated in sports (at any level) at final follow-up. Return to preinjury level of sports was not reported. Reoperations were performed in 6.9% (95% CI 4.1–9.7) of ankles and complications related to the BMS procedure were observed in 2% (95% CI 0.4–3.0) of ankles. Progression of degenerative changes was observed in 28% (95% CI 22.3–33.2) of ankles.

**Conclusion:**

Long-term clinical outcomes following arthroscopic BMS can be considered satisfactory even though one in three patients show progression of degenerative changes from a radiological perspective. These findings indicate that OLTs treated with BMS may be at risk of progressing towards end-stage ankle osteoarthritis over time in light of the incremental cartilage damage cascade. The findings of this study can aid clinicians and patients with the shared decision-making process when considering the long-term outcomes of BMS.

**Level of evidence:**

Level IV.

**Supplementary Information:**

The online version contains supplementary material available at 10.1007/s00167-021-06630-8.

## Introduction

Osteochondral lesions of the talus (OLTs) are characterised by damage to the articular cartilage and the underlying subchondral bone. Patients typically present with pain during or after weight-bearing 6–12 months after trauma, such as an ankle sprain or ankle fracture [1]. Initial treatment generally consists of non-operative management. Non-operative treatment fails in up to 55% of patients with symptomatic OLTs [2]. Operative treatment is, therefore, needed to address symptoms in the majority of these cases.

Operative management depends on lesion characteristics and patient preference in the context of shared decision-making [1, 3]. Arthroscopic bone marrow stimulation (BMS) is the most commonly performed first-line operative treatment for OLTs, and should ideally be considered for smaller (< 150 mm^2^) lesions [4, 5]. During BMS, the damaged cartilage is removed and the subchondral bone plate debrided, after which microfracturing can be performed. The goal of BMS is to initiate the formation of fibrocartilage by the release of mesenchymal stem cells and local growth factors [6]. Multiple literature studies have found that short- to mid-term outcomes of BMS could be considered acceptable and show relatively consistent clinical outcomes over time [4, 7]. However, there are conflicting outcomes of BMS concerning the clinical efficacy and clinical sustainability at long-term follow-up. The inferior quality and biomechanical properties of fibrocartilage, rather than the native hyaline cartilage, and poor subchondral bone health have been mentioned as possible reasons for the progression of osteoarthritic changes and subsequent deterioration of clinical results over time [8–10]. However, it is currently unknown on a larger group level what the long-term clinical and radiological outcomes are of arthroscopic BMS as no synthesis of the current clinical evidence nor consensus regarding its clinical efficacy exists.

It is, therefore, the purpose of the present study to assess the clinical and radiological outcomes of BMS for the treatment of OLTs at long-term follow-up. The findings of this study can improve guidance for clinicians as well as patients during the shared-decision-making process.

## Materials and methods

A systematic review of the literature was conducted. The methodology of the preferred reporting items for systematic reviews and meta-analyses (PRISMA) was followed for the conception of this study [11].

### Search strategy

The PubMed, EMBASE (Ovid), and Cochrane Library databases were searched for eligible articles. Studies from the earliest record until March 2021 were retrieved. Backwards citation chaining (i.e. reference screening) was applied during full-text screening. The full search strategy is available in Online supplementary material.

### Eligibility criteria and study selection

All studies reporting clinical outcomes of arthroscopic BMS (i.e. debridement and/or microfracturing) for OLTs at long-term follow-up were included. The inclusion and exclusion criteria are listed in Table 1. The definition of short- to long-term follow-up is subjective in clinical research, as the cutoffs vary throughout the literature [7, 12–15]. Defining a cutoff for “long-term” follow-up depends on the pathology, treatment, and patient population [16]. Long-term follow-up was, therefore, defined as a minimum of 8 years in the present study, as previously reported in the literature [17].

**Table 1:** Inclusion and exclusion criteria

Inclusion criteria	Exclusion criteria
Clinical studies reporting outcomes of arthroscopic BMS (debridement and/or microfracturing) for OLTs	Less than five patients
	No separate data for arthroscopic BMS patients available
Minimum of 8-year follow-up	Review, cadaver, and animal studies
Level I–IV peer-reviewed studies	Patient overlap
Full-text articles available in English	Asymptomatic lesions
	Level V evidence

Two authors (**Q.R.** and **J.D.**) independently conducted the title and abstract screening, as well as the full-text screening, using Rayyan [18]. When no consensus on inclusion could be reached, the senior author (**G.K.**) was decisive.

### Methodological quality

The Methodological Index for Non-Randomized Studies (MINORS) criteria were used to evaluate the methodological quality of the included studies [19]. Assessment of methodological quality was performed by two independent reviewers (**Q.R.** and **J.D.**). In case of disagreement, the senior author (**G.K.**) was decisive.

### Data extraction

Data extraction was performed by two independent reviewers (**Q.R.** and **J.D.**) using a pre-designed extraction form. Data were extracted for study characteristics (author, publication date, level of evidence, and number of patients and ankles), patient characteristics (patient age, gender, body mass index (BMI), history of trauma, duration of symptoms, follow-up time, and BMS technique (i.e. debridement or, debridement with microfracturing or drilling)), and lesion characteristics (size, lesion location, presence of cysts, and primary nature—i.e. first time surgical treatment). In addition, clinical and radiological outcomes at baseline and at follow-up were extracted. As part of the clinical outcomes, return to sport was defined according to Ardern et al. [20] as return to any level of sports and return to preinjury level of sports. If lesion location was reported according to a 9-grid scheme [21], localization was categorized according to the following distribution: medial (zone 1, zone 4, and zone 7), central, (zone 2, zone 5, and zone 8), or lateral (zone 3, zone 6, and zone 9) location. To assess signs of postoperative ankle osteoarthritis within the tibiotalar joint, a modified classification system was used, in which the Takakura et al. [22] and van Dijk et al. [23] classifications were pooled (see Table 2). The degenerative progression rate was defined as the proportion of patients who progressed with a minimum of one stage of the aforementioned modified classification system (e.g. grade 0 to grade 1 and/or grade 1 to grade 2 or higher).

**Table 2:** Modified classification of degenerative changes in the tibiotalar joint

Grade	Classification	
	Takakura [2]	van Dijk [12]
Grade 0	Undefined	Normal joint or subchondral sclerosis (van Dijk grade 0)
Grade 1	No joint space narrowing but early sclerosis and osteophyte formation (Takakura grade 1)	Osteophytes without joint space narrowing (van Dijk grade 1)
Grade 2	Narrowing of the joint space medially (Takakura grade 2)	Joint space narrowing with or without osteophytes (van Dijk grade 2)
Grade 3	Obliteration of the joint space with subchondral bone contact medially (Takakura grade 3) and, obliteration of the whole joint space with complete bone contact (Takakura grade 4)	(Sub)total disappearance or deformation of the joint space (van Dijk grade 3)

### Statistical analysis

The primary outcome was defined as clinical outcome measures consisting of pain scores, patient-reported clinical outcome measures, or physician reported clinical outcome measures. Secondary outcomes concerned the return to sport rate, reoperation rate, complication rate, and the rate of progression of degenerative changes within the tibiotalar joint. Descriptive variables were displayed as means with ranges for continuous variables and absolute numbers and frequencies for categorical variables. Due to the limited number of comparative studies and between study heterogeneity, a formal meta-analysis could not be performed. A simplified pooling method was therefore used to pool baseline characteristics and clinical outcome scores, whereby pooled means and proportions were weighted by the number of ankles per study. 95% Confidence intervals (95% CI) were calculated for pooled clinical outcome scores. 95% CI were additionally calculated using the Wilson score method (without continuity correction) [14] for the return to sport rate, reoperation rate, complications rate, and the degenerative progression rate. Ranges from the reported pooled means and proportions include the lowest and highest mean values from the included studies. Time units were converted to either weeks or months depending on the variable analysed. Lesion area was calculated in squared millimetres (mm^2^). If lesion diameter was reported, lesion size was converted into surface area using the following formula: ${\text{Area}}_{{{\text{lesion}}}} = ~\pi \,~ \times \,\left( {\frac{{{\text{lesion~diameter}}}}{2}} \right)^{2}$. Data analysis was performed in Stata 15 (StataCorp LP, College Station, TX, USA).

## Results

**Fig. 1:**
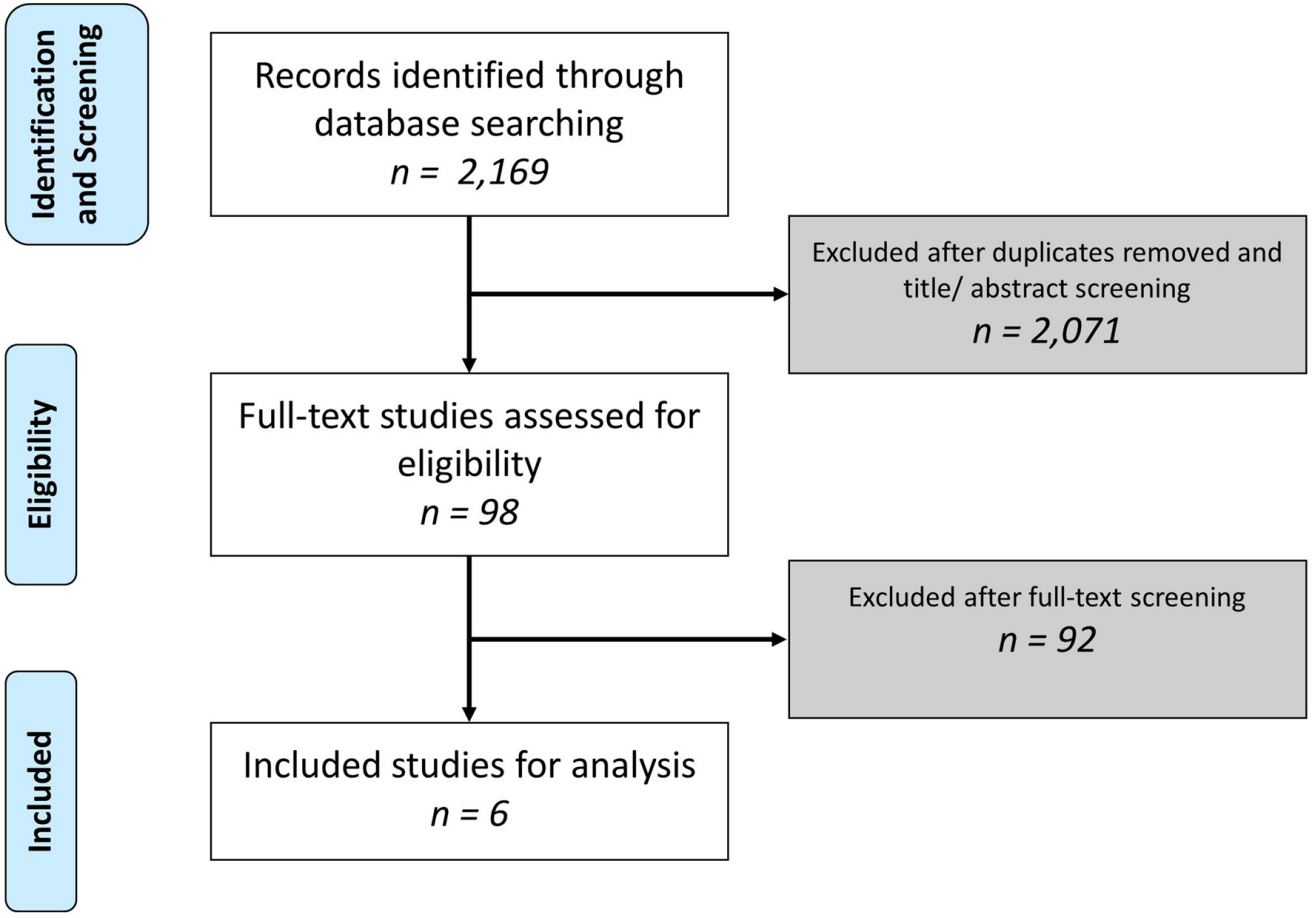
PRISMA flowchart of the study selection

A total of 2,169 records were found trough the literature search, of which 6 studies were included for final analysis (see Fig. 1) [15, 17, 24–27]. Consensus was reached for all included articles. There were two prospective cohort studies [15, 27] and four retrospective cohort studies included [17, 24–26].

### Methodological quality

Consensus was reached on the MINORS score for all included articles. The average MINORS score of the included studies was 7.7 points out of 16 (range 6–9). The MINORS score per individual study can be appreciated in Online supplementary material.

### Patient and lesion characteristics

From the 6 included studies, outcomes for a total of 310 patients (323 ankles) were reported. A full overview of the pooled baseline patient and lesion characteristics can be appreciated in Table 3.

**Table 3:** Baseline patient and lesion characteristics^a^

Patient characteristics		Percentage reported^b^ (%)
Patients, (*n*)	310	100
Ankles, (*n*)	323	100
Sex, males/females, (%)	65/35	100
Age, years (range)	37.0 (24.7–39.2)	100
Body mass index, kg/m^2^ (range)	24.7 (24.3–26.4)	69
History of trauma, (%)		86
Yes	60	
No	40	
Duration of symptoms, months (range)	10.2 (17.0–28.1)	84
Follow-up, years (range)	13.0 (9.5–13.9)	100
Method of BMS, *n* (%)		100
Debridement and microfracturing or drilling	312 (97%)	
Debridement alone	11 (3%)	
Lesion characteristics, *n* (%)		
Primary, (%)	96	94
Non-primary, (%)	4	94
Presence of cyst, *n* (%)	24	63
Lesion size		
Area, mm^2^ (range)	100.1 (74.2–105.3)	78
> 150 mm^2^	21%	63
< 150 mm^2^	79%	
Depth, mm (range)	7.1 (NA)	15
Lesion location, *n* (%)		
Medial	229 (71%)	99
Central	10 (3%)	99
Lateral	83 (26%)	99

*n* number, *BMS* bone marrow stimulation, *mm* millimetre

^a^Data are presented as weighted means (range of means) and percentages

^b^Percentage frequency of reporting by number of ankles

### Clinical outcomes

A complete overview of the clinical outcomes from the included studies is provided in Table 4. The weighted mean postoperative American Orthopaedic Foot and Ankle Society (AOFAS) score for 252 cases was 83.8 out of 100 points (95% CI 83.6–84.1).

**Table 4:** Postoperative clinical outcomes

Author, year	Ankles (*n*)	Follow-up (years)	Ogilvie-Harris	Berndt and Harty	AOFAS	VAS-pain	FOAS	Other
Baker et al. [24], (1999)	12	11.8	NA	Excellent: 5, good: 5, fair: 1, poor: 1	NA	NA	NA	NA
van Bergen et al. [17], (2013)	50	10.1	Good: 37, fair: 10, Poor: 3	Excellent: 10, good: 29, fair: 11, poor: 0	88.0 (range 64.0–100)	NA	NA	SF-36: Vitality component: 71.0 (± 16.0) Emotional component 94.0 (± 22.0)
Corr et al. [23], (2021)	45	11.6	NA		NA	14.0 out of 100 (range 0–75.0)	NA	FAAM-ADL: 90.3 (range 31.0–100) FAAM-Sports: 82.0 (range 12.5–100)
Hunt et al. [31], (2003)	8	11.8	NA	Good: 4, fair: 4, poor: 0	NA	NA	NA	Martin score: Good: 4, fair: 1, poor: 3 SANE score: Excellent: 2, good: 3, fair: 2, poor: 1
Park et al. [9], (2021)	202	13.9	NA	NA	Preop: 58.2 (± 13.6) Postop: 82.8 (± 11.7)	Preop: 7.1 out of 10 (± 1.7) Postop: 1.99 out of 10 (± 1.7)	Pain: 83.0 (± 14) Symptoms: 82.0 (± 14.6) ADL: 83.5 (± 11) Sports: 79.3 (± 11.6) QoL: 78.7 (± 12.4)	NA
Schuman et al. [38], (2002)	6	9.5	Excellent: 1, good: 4, fair: 1, poor: 0	NA	NA	NA	NA	NA

*n* number of ankles, *NA* not available, *AOFAS* American Orthopaedic Foot and Ankle Society score, *VAS* Visual Analogue Scale, *FAOS* Foot and Ankle Outcome Score, *SF*-*36* short-form 36 questionnaire, *FAAM*-*ADL* foot and ankle mobility measure—activities of daily living, *FAAM*-*Sports* foot and ankle mobility measure—sports, *SANE* single assessment numeric evaluation (SANE) question, *ADL* activities of daily living, *QoL* quality of life

Return to sport was reported in two studies [17, 25]. 68 out of 87 reported patients (78%, 95% CI 69.5–86.8) returned to any level of sports at follow-up. No specific outcomes on preinjury level of sports return were reported in any of the included studies.

### Reoperations and complications

Reoperations for OLTs were reported in five studies [15, 17, 24–26] for a total of 317 patients. At final follow-up, 22 ankles required revision surgery (6.9%, 95% CI 4.1–9.7). Reoperations following BMS consisted of repeat BMS (*n* = 12), autologous osteochondral transplantation (*n* = 6), and total ankle arthroplasty (*n* = 1). The specific type of surgical reoperation was not reported for three patients.

Complications related to the BMS procedure were reported for 260 cases in a total of 3 studies [15, 17, 26]. Among the reported group of cases, four complications were observed (2%, 95% CI 0.4–3.0)—all were due to neurological complications related to arthroscopy.

### Radiological outcomes

Three studies reported radiological outcomes at final follow-up [15, 17, 27]. All studies assessed postoperative degenerative changes within the tibiotalar joint using radiographs. Two studies [17, 27] used the van Dijk score [23] and one study [15] reported the Takakura score [22]. From the 256 ankles assessed at final follow-up, the following osteoarthritic stages were reported: grade 0 in 168 ankles (66%), grade 1 in 77 ankles (30%), grade 2 in 10 ankles (3%), grade 3 in 1 ankle (1%). Progression of degenerative changes was reported for 71 out of 256 ankles, with a corresponding degenerative progression rate of 28% (95% CI 22.3–33.2).

## Discussion

The most important finding of the present study is that long-term clinical outcomes of arthroscopic BMS for primarily smaller (< 150 mm^2^) lesions are satisfactory. This important outcome is necessary to evaluate in the light of limited data due to heterogenous reporting of data and low-level of evidence. Progression of degenerative changes was found in approximately one out of three patients.

Clinical outcomes of BMS for OLTs have been reported to be good to excellent at short- to mid-term follow-up [4, 7]. Toale et al. [7]—who reviewed the literature on clinical outcomes of 858 ankles treated with BMS for primary OLTs at mid-term follow-up—found that clinical outcomes remain adequate. The aforementioned study reported a pooled AOFAS score of 89.9 points and a reoperation rate of 6% at a mean 6-year follow-up. Previously, no study has specifically pooled evidence for long-term clinical outcomes of BMS. When comparing the clinical results of the present study to the findings of Toale et al. [7], a slight decrease in AOFAS score, though comparable revision rate, can be observed. The rationale for specifically investigating outcomes at long-term follow-up lies in the hypothesis that BMS may not be a sustainable treatment option for all OLTs [7, 12, 17, 28]. This may be due to the development of degenerative changes within the repair tissue and tibiotalar joint [7, 17, 28]. A number of reasons for the degeneration of repair tissue after BMS and consecutive clinical failure have been proposed in the literature. First, it is known that lesion filling by fibrocartilage shows inferior wear characteristics compared to the native hyaline cartilage [29, 30]. Fibrocartilage predominantly consists of type-1 collagen which is biomechanically inferior to type-2 collagen that is primarily expressed in hyaline cartilage [30]. This hypothesis may be substantiated by findings from second-look arthroscopy, which show a poor quality of the cartilage repair tissue [31]. Second, fibrocartilage may not be able to sufficiently protect the underlying subchondral bone, which is an important structure for cartilage health and the load-bearing capacity of the talus, and thus plays an important role in the development of osteoarthritis [28, 32]. Reilingh et al. [9] found that 74% of patients show a depressed subchondral bone plate at one-year after the initial surgery. Moreover, Shimozono et al. [10] found that the subchondral bone degradation is associated with lower clinical outcomes after BMS at mid-term follow-up. These findings drive the hypothesis that repair tissue degradation and osteoarthritis formation play an important role in deteriorating clinical outcomes. When assessing the incidence of degenerative changes as a measure for ankle osteoarthritis in the present study, one in three patients were found to show radiographic degenerative changes at final follow-up. However, it should be mentioned that the majority of these patients showed osteophyte formation or minimal degenerative changes and a limited proportion of patients presented with joint space narrowing and/or total joint obliteration (i.e. late-stage ankle osteoarthritis). Caution should be warranted for the interpretation of these findings as this study concerns a low number of patients. The findings of degenerative changes observed in the present study concur with previous studies on long-term outcomes of OLT treatment in which a high rate of early-stage degenerative changes were observed but a low proportion of patients was found to have end-stage ankle osteoarthritis [33–35]. When examining long-term outcomes of cartilage lesions in the knee, a high degree of patients is observed to develop osteoarthritis after sustaining a cartilage lesion and undergoing BMS [36–38]. The progression of degenerative changes in the knee is associated with poor clinical outcomes following BMS [36, 37].

Inferior wear characteristics of talar fibrocartilage and poor subchondral bone health may be exacerbated by an increased lesion size [28]. It has been well established that lesion size is an important factor for the clinical success of BMS in OLT treatment. Current evidence from clinical studies shows that the optimal cutoff for clinical outcomes after BMS is between 110 and 150 mm^2^ (10–15 mm diameter) [5, 39, 40]. When specifically considering this in the context of long-term follow-up as assessed in the present study, it becomes clear that 160 out of 202 (79%) available patients were reported to have an OLT smaller than 150mm^2^. These data were available for a single study, by Park et al. [15]. The aforementioned study reported that a lesion size area of ≥ 150mm^2^ and a BMI of ≥ 25 was associated with a significantly higher reoperation rate for the 12 (out of 202) failed procedures. In contrast, van Bergen et al. [17] and Corr et al. [25] did not find a significant association between lesion size and clinical outcomes. This may be attributable to the lower number of patients included in both studies. The findings of the present study show large long-term (prognostic) database studies are highly needed to elucidate the influence of prognostic factors such as lesion size on the survivability of BMS procedures.

When assessing the sports participation rate of patients included in the present study it is important to note that no return to sport participation level (i.e. return to preinjury—or any level of sports [41]) nor preinjury level of sports (i.e. recreational, competitive, or professional athlete) was reported. All patients were, therefore, classified as having returned to any level of sports. Lambers et al. [13] found that 90% of patients participated at any level of sports after BMS at a mean 6.4-year follow-up. However, it was noted in the aforementioned study that 53% of patients were able to return to their preinjury level of sports. Steman et al. [41] observed that patients treated with BMS were able to return to any level of sports in 88% of the cases and to a preinjury level of sports in 79% of the cases. It may therefore be the case that sports participation after BMS decreases over time, especially when considering preinjury level of sports. However, the results of the present study are limited to a low number of cases and are at risk of bias. More research on long-term sports participation after BMS is highly necessary. It is crucial that future studies report sport outcomes based on the level of preinjury and any level of sport participation. These outcomes are of importance for assessing the long-term effects of any OLT treatment and for the shared decision-making between physicians and patients.

The present study is not without its limitations. First, the included articles were of low-level of evidence. In addition, a limited number of patients were available for analysis and data were heterogeneously reported. Moreover, the study by Park et al. [15] made up the majority of included patients assessed from the included studies, which could introduce bias. Due to the underreporting and heterogenous reporting of outcomes, it was challenging to pool clinical outcomes. The only clinical outcome which could be pooled was the AOFAS score. The AOFAS score is, however, regarded as a subjective scoring system and is not validated for the use in patients with an OLT. Future studies should, therefore, focus on including validated clinical outcome measures. Second, the present study included both primary and non-primary lesions as well as differing lesion morphologies (i.e. lesion area, lesion depth, and the presence of cysts) which could affect outcomes and introduce bias. The outcomes of the present study concern a heterogenous patient group and caution is warranted when interpreting the findings of this study. A strength of the present study was that the presence of degenerative changes in the tibiotalar joint was assessed prospectively in all available studies [15, 17, 27]. It is highly necessary to prospectively follow-up patients at predetermined time points to increase the level of evidence for BMS outcomes at long-term follow-up and optimise the treatment indication.

The clinical relevance of this study concerns the clinical and practical summary of current available evidence for BMS and the conclusion that BMS yields satisfactory clinical outcomes at long-term follow-up. The findings of the study can aid clinicians and patients in the shared decision-making process when considering the long-term outcomes of BMS in the context of an individualised treatment plan. Another clinical application of the present study is the identification of a clear research gap within OLT treatment and recommendations for future research concerning the long-term clinical outcomes of BMS.

## Conclusion

Long-term clinical outcomes following arthroscopic BMS can be considered satisfactory even though one in three patients show progression of degenerative changes from a radiological perspective. These findings indicate that OLTs treated with BMS may be at risk of progressing towards end-stage ankle osteoarthritis over time in light of the incremental cartilage damage cascade. The findings of this study can aid clinicians and patients with the shared decision-making process when considering the long-term outcomes of BMS.

## Supplementary Information

Below is the link to the electronic supplementary material.

Supplementary file1 (DOCX 25 KB)

## Abbreviations

OLT: Osteochondral lesion of the talus
BMS: Bone marrow stimulation
MINORS: Methodological index for non-randomised studies
95% CI: 95% Confidence interval
AOFAS: American orthopaedic foot and ankle society
