# Supplementary material for: Satisfactory long-term clinical outcomes after bone marrow stimulation of osteochondral lesions of the talus
Source: Knee Surg Sports Traumatol Arthrosc. 2021 Jun 29;29(11):3525–33. doi: 10.1007/s00167-021-06630-8 (PMC8514351; doi:10.1007/s00167-021-06630-8)
Supplement: Supplementary file 1 — Supplementary file1 (DOCX 25 KB) [file 167_2021_6630_MOESM1_ESM.docx]

# Appendix

**Appendix 1:** Search Strategy

| **PUBMED** | **Search terms** |
| --- | --- |
| #1 | "Osteochondritis Dissecans"[Mesh] |
| [#](https://www.ncbi.nlm.nih.gov/pubmed/advanced)2 | Osteochondritis dissecans[tiab] OR osteochondrosis dissecans[tiab] OR osteochondrolysis[tiab] OR OCD[tiab] OR OLT[tiab] |
| #3 | (osteochondral[tiab] OR chondral[tiab] OR transchondral[tiab] OR cartilage*[tiab]) AND (defect*[tiab] OR lesion*[tiab]) |
| #4 | #1 OR #2 OR #3 |
| #5 | "Talus"[Mesh] |
| #6 | talus[tiab] OR talar*[tiab] OR ankle[tiab] |
| #7 | #5 OR #6 |
| #8 | #4 AND #7 |
| **EMBASE** | **Search terms** |
|  | (osteochondritis dissecans/or (osteochondritis dissecans or osteochondrosis dissecans or osteochondrolysis or OCD or OLT).ti,ab,kw. or ((osteochondral or chondral or osteochondral or transchondral or cartilage*) adj3 (defect* or lesion*)).ti,ab,kw.) and (talus/ or (talus or talar* or ankle).ti,ab,kw.) |
| **COCHRANE** | **Search terms** |
| #1 | MeSH descriptor: [Osteochondritis Dissecans] explode all trees |
| #2 | osteochondritis dissecans or osteochondrosis dissecans or osteochondrolysis or OCD or OLT:ti,ab,kw (Word variations have been searched) |
| #3 | (osteochondral or chondral or transchondral or cartilage*) and (defect* or lesion*):ti,ab,kw (Word variations have been searched) |
| #4 | #1 or #2 or #3 |
| #5 | MeSH descriptor: [Talus] explode all trees |
| #6 | Talus or talar* or ankle*:ti,ab,kw (Word variations have been searched) |
| #7 | #5 or #6 |

| **Appendix 2:** Minors criteria of included studies | | | | | | | | | Additional criteria comparative research | | | |  |
| --- | --- | --- | --- | --- | --- | --- | --- | --- | --- | --- | --- | --- | --- |
| Study | A clearly stated aim | Inclusion of consecutive patients | Prospective collection of data | Endpoint appropiate to the aim of the study | Unbiased assesment of the study endpoint | Follow up period appropiate to the aim of the study | Lost of follow up less than 5% | Prospective calculation of study size | An adequate control group | Contampory group | Baseline equivalent of groups | Adequate statistical analysis | Total |
| van Bergen, 2013 | 1 | 2 | 0 | 2 | 1 | 2 | 0 | 0 |  |  |  |  | 8/16 |
| Baker, 1999 | 1 | 2 | 0 | 2 | 1 | 2 | 0 | 0 | - | - | - | - | 8/16 |
| Corr, 2021 | 1 | 2 | 0 | 2 | 1 | 2 | 0 | 0 | - | - | - | - | 8/16 |
| Hunt, 2003 | 1 | 1 | 0 | 2 | 1 | 2 | 0 | 0 |  |  |  |  | 7/16 |
| Schuman, | 0 | 2 | 0 | 1 | 1 | 2 | 0 | 0 |  |  |  |  | 6/16 |
| Park. 2021 | 1 | 2 | 0 | 2 | 1 | 2 | 1 | 0 |  |  |  |  | 9/16 |
